# Motivating Adherence to Exercise Plans Through a Personalized Mobile Health App: Enhanced Action Design Research Approach

**DOI:** 10.2196/19941

**Published:** 2021-06-02

**Authors:** Ruo-Ting Sun, Wencui Han, Hsin-Lu Chang, Michael J Shaw

**Affiliations:** 1 Department of Business Administration University of Illinois, Urbana-Champaign Champaign, IL United States; 2 Department of Management Information Systems National Chengchi University Taipei Taiwan

**Keywords:** adherence, mobile health, motivation, personality, MBTI, action design research, mobile phone

## Abstract

**Background:**

Physical inactivity is a global issue that affects people’s health and productivity. With the advancement of mobile technologies, many apps have been developed to facilitate health self-management. However, few studies have examined the effectiveness of these mobile health (mHealth) apps in motivating exercise adherence.

**Objective:**

This study aims to demonstrate the enhanced action design research (ADR) process and improve the design of mHealth apps for exercise self-management. Specifically, we investigate whether sending motivational messages improves adherence to exercise plans, whether the motivational effect is affected by personality, the impact of message type and repetition, and the process of involving a field experiment in the design process and learning new design principles from the results.

**Methods:**

This formative research was conducted by proposing an enhanced ADR process. We incorporated a field experiment into the process to iteratively refine and evaluate the design until it converges into a final mHealth app. We used the Apple ResearchKit to develop the mHealth app and promoted it via trainers at their gyms. We targeted users who used the app for at least two months. Participants were randomly assigned to 1 of the 12 groups in a 2×3×2 factorial design and remained blinded to the assigned intervention. The groups were defined based on personality type (thinking or feeling), message type (emotional, logical, or none), and repetition (none or once). Participants with different personality types received tailored and repeated messages. Finally, we used the self-reported completion rate to measure participants’ adherence level to exercise plans. By analyzing users’ usage patterns, we could verify, correct, and enhance the mHealth app design principles.

**Results:**

In total, 160 users downloaded the app, and 89 active participants remained during the 2-month period. The results suggest a significant main effect of personality type and repetition and a significant interaction effect between personality type and repetition. The adherence rate of people with feeling personality types was 18.15% higher than that of people with thinking types. Emotional messages were more effective than logical messages in motivating exercise adherence. Although people received repeated messages, they were more likely to adhere to exercise plans. With repeated reminders, the adherence rates of people with thinking personality types were significantly improved by 27.34% (*P*<.001).

**Conclusions:**

This study contributes to the literature on mHealth apps. By incorporating a field experiment into the ADR process, we demonstrate the benefit of combining design science and field experiments. This study also contributes to the research on mHealth apps. The principles learned from this study can be applied to improve the effectiveness of mHealth apps. The app design can be considered a foundation for the development of more advanced apps for specific diseases, such as diabetes and asthma, in future research.

## Introduction

### Background

In modern society, many people live a fast-paced, high-stress lifestyle and do not engage in regular physical activity. A study showed that 80% of adults in America lack physical exercise [1]. A lack of exercise is the main cause of most chronic diseases [2] and the leading cause of death worldwide [3,4]. Research also suggests that regular exercise improves the mood and self-reported performance of white-collar workers [5]. With the advancement of mobile and wearable technologies, many mobile health (mHealth) apps have been developed to enhance exercise adherence and facilitate a healthy lifestyle. mHealth apps are defined as “mobile applications that assist consumers in self-management of overall wellness, disease prevention, and disease management” [6]. The mHealth app market has been steadily growing over the past few years. According to an industry report [7], more than 318,000 mHealth apps are available in the top app stores worldwide, and more than 200 apps are added daily.

Despite the popularity of mHealth apps, questions remain regarding their sustained effectiveness. According to a recent survey [8], 87% of patients used mHealth apps, but more than one-quarter of them stopped using these apps because of ineffectiveness in helping achieve their health goals or yielding tangible results. Prior research has also noted that many mHealth apps lack theory-based motivational techniques, rendering it difficult for these apps to sustain over the long term [9]. Moreover, mHealth apps are often designed and treated as *black boxes*; the design is not evidence-based [10,11]. These findings suggest that mHealth app developers must design tools to be more engaging for users. However, few studies have examined the quality of mHealth apps in terms of their effectiveness in motivating health-related behaviors or the design principles of these apps from the user perspective [12-14].

### Prior Work

#### Overview

Previous research discussed the optimal means of designing and evaluating persuasive systems and suggested a set of persuasive principles to design and evaluate these systems [15]. Persuasive principles are specific design techniques such as providing reminders, tailored and personalized information, *social support*, or suggestions. By including persuasive principles, mHealth apps can more effectively motivate users to adhere to their plans [16]. For example, a study conducted by Middelweerd et al [17] reviewed 64 of 41,246 mHealth apps available on Google Play and iTunes stores and suggested that techniques such as self-monitoring, receiving feedback on performance, and setting goals were most frequently used for persuasion. A review of mHealth apps that applied persuasive technology to improve physical exercise indicated that users were persuaded and became more involved in disease control or health management when persuasive design principles were applied [18]. Informed by the literature, in this study, we apply 3 persuasive design principles when designing an app and examined their effectiveness in motivating exercise. These principles included (1) tailored communication, (2) motivational messages, and (3) repetition of messages.

#### Tailored Communication

Past literature has suggested that the impact of health communication is generally enhanced when it is tailored to a specific individual [19]. However, the principles of audience segmentation are far less discussed in health care than in advertising, marketing, and social marketing [20,21]. In early work in this field, segmentation was often based on demographic differences. For example, different self-help guides for smoking cessation have been designed for blue-collar and minority smokers [22], African Americans [23], older smokers [24], pregnant women [25], and women with young children [26]. However, few studies have investigated the effectiveness of health communication by using *personality type* as a central tailoring variable. In this study, we followed the psychological type theory [27] and the Myers-Briggs Type Indicator (MBTI) test [28] to specifically consider 2 personality types, namely, the thinking type and the feeling type. The psychological type theory suggests that a person’s seemingly random behavior is based on their inner preferences regarding perceiving and organizing information to form conclusions. The MBTI is a self-report questionnaire that can be used to assess people’s psychological preferences in perceiving the world and making decisions. The MBTI is a well-adopted method used to quantify psychological types and is widely used in empirical studies to measure people’s decision-making behavior [29-33]. The MBTI assesses personality types by considering a person’s preference based on the following 4 pairs of psychological types: extraversion and introversion assess how people direct their energy either outwardly toward people and activities or inwardly toward thoughts and ideas; sensing and intuition refers to two ways of gathering information and understanding situations; thinking and feeling are two ways in which people organize and structure information and draw conclusions; and finally, judging and perceiving describes how people prefer to live their outer life. By adapting the MBTI, we were interested in investigating the impact of personality on the effectiveness of mHealth apps in promoting adherence to exercise plans. Therefore, thinking and feeling were selected as this pair represents the key dimension measuring how people organize and structure information and make decisions. People with a thinking personality type prefer applying analytical and logical principles to make objective decisions by following clear and consistent principles, whereas people with a feeling personality type may opt to make decisions by referencing their own and others’ values and place more weight on personal concerns.

#### Motivational Messages

Several studies have investigated the effectiveness of motivational or persuasive messages in promoting targeted behaviors in the fields of psychology [34,35], marketing [36,37], public health [38,39], and health management [40-43]. Research has examined emotional versus rational messages [36,44,45], type of elaboration [37], positive versus negative messages, gain versus loss framing [39-41,46,47], and source credibility and likability [34]. The results of these studies consistently show that persuasive messages promote intended behaviors. In this research, we were specifically interested in the effect of messages that generate positive emotions (*emotional* messages) compared with that of messages based on facts (*logical* messages). According to the Toulmin model of argumentation [48] and research conducted by Kim and Benbasat [49], a good argument with grounds (data), claims, and warrants leads to the highest level of trusting belief. We define logical messages as logical arguments consisting of a claim, data (ie, facts that support the claim), and backing (ie, data credibility). For example, “Don’t forget to exercise today! Research shows that even one session of exercise will enhance your positive mood.” The claim “Don’t forget to exercise today,” “even one session of exercise will enhance your positive mood” represents the data, and “research shows” provides the backing. In contrast, research has also shown that manipulating emotions accompanying a persuasive message affects the effectiveness of the message. People tend to adjust their beliefs to fit their emotions as people treat feelings as evidence [50]. Emotional stimuli can influence judgments without the judge’s awareness of such stimuli [51]. In contrast to logical messages, emotional messages do not provide facts to support the claim but focus on triggering positive emotions (ie, claims plus positive emotion stimuli). For example, “It’s time for your exercise! You are doing a fabulous job!”

#### Repetition of Messages

In addition to the message content, previous research has examined the impact of technical features of messages, such as message length [36], position [40,52], and repetition [37,53]. Among all the features, the most relevant and customizable feature in our context is repetition. The effect of repetition is referred to as the *mere repeated-exposure effect* in sociology, a psychological phenomenon involving people’s tendency to develop a preference merely because they are repeatedly exposed to something [54]. A vast body of literature has shown that the repeated-exposure effect is a robust phenomenon demonstrated across cultures and diverse stimulus domains [55-60]. There are many applications of the mere repeated-exposure effect. In marketing, for example, many studies have tested the effects of advertisement repetition [61-63]. The repeated-exposure effect has also been studied in many other social and human decision-making contexts [62,64-66]. For example, in a study related to reminders using computer systems, Malone [67] suggested that displaying high-priority tasks more frequently is an effective reminder strategy. Therefore, we were interested in investigating the impact of message repetition on exercise adherence.

### The Goal of This Study

This study’s purpose was to improve the design of mHealth apps for exercise self-management by using an innovative research approach that combines field experiments with action design research (ADR). The literature examining adherence to exercise plans has defined and measured *adherence* as the percentage of completion of an exercise plan—often pertaining to plans agreed upon by patients and care providers in the case of medical studies [68,69]. We adopted this definition and measurement method by defining adherence to an exercise plan as the percentage of the exercise plan completed, and adherence was used as the dependent variable in this study. Notably, in this study, participants established their own plans. As they had very different health conditions and physical capabilities, it was not realistic for the researcher to create a universal plan. As the study participants likely had the desire to exercise, they set goals that they deemed beneficial to their health.

Specifically, our research objectives included (1) investigating whether sending motivational messages could improve adherence to exercise plans, (2) considering whether the motivational effect was impacted by personality, (3) testing message type (logical vs emotional) and repetition impact, and (4) exploring the possibility of involving a field experiment in the design process, learning new design principles from the results.

## Methods

### Overview

We followed an ADR approach to conduct this study. ADR, proposed by Sein et al [70], conceptualizes the research process as comprising the inseparable and inherently interwoven activities of building the information technology artifact, intervening in the organization, and concurrently evaluating the artifact. The process includes the following stages:

- Stage 1: problem formulation. In this stage, researchers and stakeholders determine the initial scope, decide the roles and scope of practitioner participation, and formulate the initial research questions.
- Stage 2: building, intervention, and evaluation. In this stage, based on the problem framing and theoretical premises adapted in stage 1, the research team builds the initial information technology artifact. The process should be performed as an iterative process in a targeted environment.
- Stage 3: reflection and learning. The reflection and learning stage proceeds conceptually from building a solution for a particular scenario to applying learning to a broader class of problems.
- Stage 4: formalization of learning. This stage generalizes the principles learned to a class of field problems.

**Figure 1:**
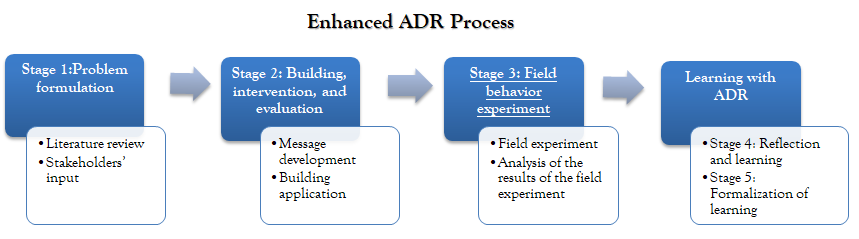
Research process—enhanced action design research process. ADR: action design research.

To examine the effectiveness of an mHealth app in promoting adherence to an exercise plan, we enhanced the ADR process by adding a field experiment step. In this stage, we used artifacts to examine the specific design principles. By understanding how users interact with the artifact, we were able to link the behavior study with design research and then verify and correct the design principles. We demonstrate the research process in Figure 1 and detail the process in the following section.

### Stage 1: Problem Formulation

On the basis of a literature review, review of web-based comments for mHealth apps, and focus groups with users, we identified mHealth apps as lacking theoretical guided design and found that few mHealth apps are focused on motivation, which is one of the main problems, along with the lack of evaluation of effectiveness, as detailed in the *Introduction* section. We identified app developers, users, and health care professionals (ie, gym trainers in our research settings) as stakeholders. We recruited 3 gym trainers and their trainees during the process of forming our initial research scope. While working with the researchers (among them, a participant was also the developer of the research app), all stakeholders provided input during the process. The gym trainers contributed their thoughts based on their prior experience with exercise management, that is, persuasive principles, such as daily progress reports of diet and exercise plans to supervise performance of trainees. We also interviewed trainees to learn about their prior experience using mHealth apps and summarized several necessary functions, such as event schedulers, alarm reminders, and progress reports. By understanding the mechanisms motivating the trainees, we and the developer discussed the executability of certain mHealth functionalities and how to maximize the effectiveness of motivational messages. The stakeholders can benefit from using our designed mHealth app as a tool to monitor and manage their exercise plans and learn persuasive principles. We defined the scope of the project as follows: develop an mHealth app to remind and motivate users to adhere to their exercise plans and examine the impact of personality and message characteristics on adherence. We intended to generate design principles for health self-management and other motivational applications.

### Stage 2: Building, Intervention, and Evaluation

#### Message Selection

On the basis of the definition of logical and emotional messages detailed in the *Introduction* section, we selected and edited motivational messages from web-based forums. We first selected several popular sites where users post motivational messages related to exercise and fitness; then, messages that were clearly emotional or logical were manually selected. Next, we ensured that the messages selected were shorter than 128 characters to fit into a text message. We used lab testing to validate the message type classification, as described in the following sections.

#### Message Type Cross-validation

To classify the messages into emotional and logical types, we conducted 2 rounds of tests. Each round included 5 judges, who were students and faculty members in an information systems department and were not involved in the project. In the first round, the judges first read the definitions of logical and emotional messages and then classified each message as either an emotional or logical message. Any message that was not correctly classified by all the judges was removed from the message pool; we retained 30 logical and 30 emotional messages after the first round of classification. In the second round, we had another 5 judges to classify retained messages.

The interrater reliability measured by Cohen κ statistics was higher than 0.86 for any pair of judges (no more than 1 disagreement). After the 2 rounds of tests, we decided that the reliability of the classification of the messages had met the standards [71], so we used the messages in the following steps.

#### Pretest of the Message Impact

To pretest the impact of motivational messages, we recruited 100 college students to participate in pretesting. The participants took the MBTI test to determine whether they had a thinking or feeling personality type. Participants then read the motivational messages selected in the previous step and rated the perceived motivational level of each message on a 7-point Likert scale (strongly disagree to strongly agree with the effects of the motivational messages).

The average rating (mean 5.8, SD 1.6) of all messages suggested that participants found the messages motivational. Participants with a feeling personality type rated both emotional and logical messages higher than participants with a thinking personality type. The messages used in the final field experiment are presented in Multimedia Appendix 1.

#### Building the App

On the basis of the literature review and theoretical background detailed in the previous section, we envisioned an effective mHealth app that could set up alarm reminders for exercise (ie, message repetition) and send personalized motivational messages (ie, emotional or logical messages) based on users’ personality types (ie, thinking type or feeling type). We used the Apple ResearchKit to develop an mHealth app named ActiveTrack. The idea behind the Apple ResearchKit is for scientists and drug developers to *build mobile or wearable apps* that suit their particular needs, whether for the collection of research data, patient recruitment, or the collection of informed consent. A prototype of the app was built with the initial design and released to the Apple app store, and user activity data were saved in a web-based database. By observing app users’ usage patterns, we identified points at which users were more likely to stop using the app. We also recruited users who provided feedback about the design and adjusted it based on these inputs. For example, in the initial design, users had to answer survey questions that collected their information, such as demographics, medical histories, and the MBTI questions, before entering the main page of the app. We observed that this caused most users to stop using the app. In total, 35 of the 79 inactive users dropped out at the beginning stage because of the overwhelming questions. Therefore, we reduced the number of questions and made the process shorter. We also made setting up reminders easier.

In the beta cycle, more users used ActiveTrack. Owing to the adjustments made, these users remained active for a longer time than the initial users. We hosted focus groups with some users and incorporated more suggestions into the design.

#### Design Artifact

The mHealth app (ActiveTrack) finalized for the field test offered the following features and materials (screenshots are provided in Multimedia Appendix 2):

- *Study information and participant consent pages*. This page provided the basic information of the study; the user could indicate their consent for participation in the study at the end of the page.
- *Survey of participants’ demographic information, living situation, initial motivation level, and exercise habits*. This section allowed users to skip any question they did not want to answer.
- *MBTI survey*. This page provided a questionnaire to determine personality type.
- *Exercise plans and alarms*. Users could enter any number of exercises they wanted to perform every day, such as running for 10 minutes at 6 AM and running for 15 minutes at 6 PM. They could also decide the number of days they wanted to exercise per week. We allowed users to set their goals to ensure that they were tailored to their health conditions. We defined adherence to exercise plans as adherence to users’ own plans instead of a universal plan, as, in reality, users have very different health and living conditions, and it is not realistic to establish a universal plan.
- *Display of motivational messages* (settings vary based on the experimental groups, as described in the *Field Experiment* section). An alarm would ring at the time the user set for exercise; a message would be pushed to the main screen (unless the user was in a group that did not receive messages). The alarm could be snoozed 3 times for 5 minutes each. For the groups with repetition, the first notification (with or without a message) appeared 30 minutes in advance to remind users of their plans; then, the reminder appeared again at scheduled times.
- *Record of the exercise plan was achieved*. The app asked whether the exercise plans were followed a few hours after the scheduled time, and users could click *yes* or *no* for each exercise item.

### Stage 3: Field Experiment

In this research, we designed a field experiment and received approval from the institutional review board of the University of Illinois. We conducted a field experiment using the mHealth app ActiveTrack. ActiveTrack is an exercise planning mobile app designed based on our theory-based message tailoring method that aims to examine the effects of (1) tailored communication, (2) motivational messages, and (3) repetition of messages on users’ adherence to their goal settings. ActiveTrack was available to anyone for download from the Apple store. We also promoted our app through gyms, where many members participated in weight loss programs. Our partnered trainers introduced ActiveTrack to their trainees but were not authorized to monitor their trainees’ behavior on the app. We targeted users who used the app for at least two months. The experiment included the following steps:

- Participants downloaded the research app (the user needed to have an iPhone).
- Participants were randomly assigned following simple randomization procedures (computerized random numbers) to 1 of 12 groups when they downloaded the app (randomization was achieved by embedding the random assignment process in the app development). The groups were defined as follows:
  personality type: thinking type or feeling type
  message type: no message, emotional message, or logical message
  repetition: not repeated or repeated once (ie, 1 reminder 30 minutes before the scheduled alarm)

Therefore, we had a 2×3×2 design with 12 groups. Participants remained blinded to the assigned intervention to prevent them from being influenced by such knowledge;

- Participants were presented with the study’s information (ie, purpose, procedures, voluntary nature, confidentiality, risks and benefits, and contact information) and signed a consent form (the declaration can be found in Multimedia Appendix 3).
- Participants provided background information such as age, sex, race, weight, and exercise times per week through the app (the information page is available in Multimedia Appendices 2 and 4).
- Participants completed the MBTI questions through the app to allow us to determine their personality types. As we focused on only one dimension of the MBTI test, as described in the *Tailored Communication* section, we selected only the questions that could determine a person’s thinking or feeling personalities (MBTI assessment in Multimedia Appendices 2 and 5).
- Participants entered their exercise plans (open-ended text entry) and alarms (specific time). Exercise could be performed any number of times per week and per day based on individual health conditions. Example screenshots can be found in Multimedia Appendix 2.
- Participants received tailored messages. On the basis of our 2×3×2 research design, there were 12 scenarios. Participants with thinking or feeling personality types may receive emotional, logical, or no messages with or without message repetition. Examples of the message display can be found in Multimedia Appendix 2.
- Participants used the app and self-reported whether each exercise item was completed (completion rate). Example screenshots are shown in Multimedia Appendix 2.
- All user inputs, alarms, and reminders were recorded in a web-based database. For privacy protection, we did not collect information that could be used to identify users.

## Results

### Participants

In total, 160 users downloaded the app during the 2-month period; after excluding users who stopped using the app in the middle of the process, 89 participants remained. The attrition rate was 44.4% (71/160). We compared the characteristics of those who dropped out (inactive users) and those who remained (active users) and did not find any significant differences. Of these active participants, 55 were female and 31 were male (3 did not indicate their sex). Participants’ ages ranged from 19 to 56 years, with a mean age of 28.8 (SD 8.3) years. Among all active users, results showed no significant difference in adherence level across sexes and ages. In addition, we found that white-collar workers had 17% higher adherence level to exercise plans than blue-collar workers (*t*_63_=−2.045, two-tailed; *P*=.04). Moreover, higher exercise frequencies per week were associated with a higher adherence level (*t*_63_=2.341, two-tailed; *P*=.02). We further tested the difference in adherence between those who downloaded the app following their trainers’ recommendations and those who downloaded the app voluntarily, and the results showed no significant differences between the 2 groups (*t*_63_=−0.254, two-tailed; *P*=.80).

We also compared the characteristics of those who dropped out (inactive users) and those who remained (active users). We found no significant difference in age and exercise frequency between the active and inactive groups. We used a chi-square test to examine any differences between active and inactive users in sex, job type, and working hours. There were no significant differences in sex and working hours between the 2 groups. However, in the active group, we found that white-collar workers had a higher adherence rate than blue-collar workers. In the inactive group, white-collar workers were more likely to quit. The job type was a critical factor that affected users’ retaining behavior.

The sample size was relatively small because of the difficulty of recruiting active users; however, the sample size was similar to that in research in the health care domain, which investigated the effectiveness of using mobile phones for health management. In a review of the effectiveness of mHealth and technology-based health behavior management interventions, all 7 studies related to physical activity behaviors included 17-150 participants [72]. Payne et al [13] systematically searched and described the literature on mobile apps used in health behavior interventions; 17 of the 24 studies reviewed had a sample of fewer than 100 participants. In addition, there was a limited impact of the low power caused by the small sample size in this study, as detailed in the following sections. The number of active participants in each group and the mean and SD of plan achievement are presented in Table 1.

**Table 1:** Descriptive statistics of the active participants in each group.

Personality and message type		Message repetition	Adherence rate, mean (SD)	Active participants, n (%)
**Thinking**				
	**None**			
		None	41.67 (45.78)	12 (13)
		Once	77.31 (29.72)	14 (16)
	**Logical**			
		None	57.51 (46.10)	7 (8)
		Once	92.99 (14.54)	9 (10)
	**Emotional**			
		None	73.80 (17.18)	11 (12)
		Once	86.58 (16.40)	11 (12)
**Feeling**				
	**None**			
		None	83.33 (28.87)	3 (3)
		Once	91.67 (16.67)	4 (4)
	**Logical**			
		None	95.24 (8.25)	3 (3)
		Once	83.73 (21.10)	6 (7)
	**Emotional**			
		None	91.75 (10.45)	3 (3)
		Once	94.21 (9.48)	6 (7)

### Model Testing Results

#### Overview

**Figure 2:**
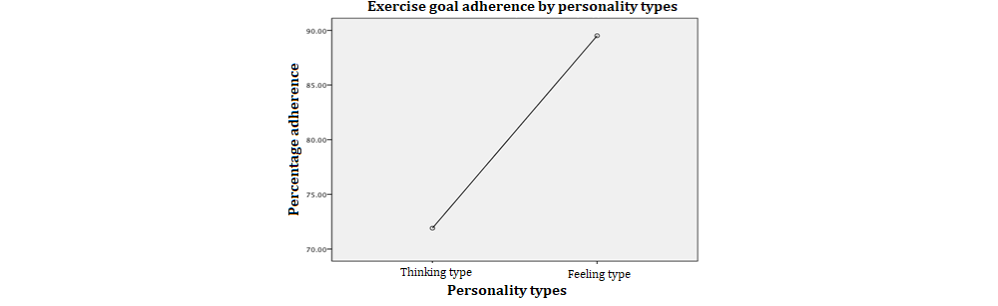
Personality type main effect.

**Figure 3:**
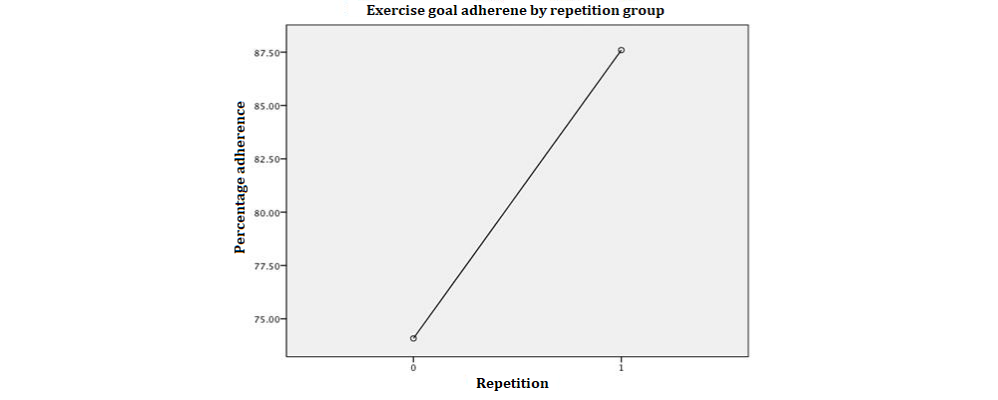
Repetition main effect.

**Figure 4:**
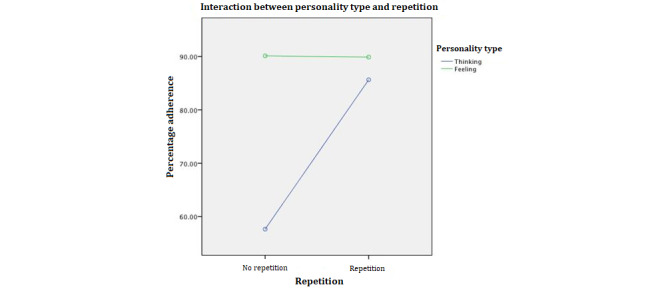
Interaction effect of personality type and repetition.

To examine the effects of personality type, message type, and repetition of reminders as well as the two-way interaction effects between the variables on adherence to exercise plans, we used a three-way analysis of variance. To meet the assumption of homogeneity of variance of error, we used a square operation to transform the dependent variable. The *F* test for heteroskedasticity suggests that the equal variance of the error assumption is met (*F*_1,85_=1.26; *P*=.26). The results of the analysis of variance model are presented in Table 2. The results suggested a good overall fit of the main effects of personality type and repetition and a significant interaction between personality type and repetition. To understand the main effects, we conducted a Tukey honest significant difference posthoc analysis to compare the mean difference across the different groups; the results are presented in Table 2 and Figures 2-4.

**Table 2:** Model testing results.^a^

Source	Type III sum of squares	Mean square	*F* test (*df*)	*P* value
Personality type	73,471,244	73,471,244	6.537 (1)	.01
Message type	32,216,925	16,108,463	1.433 (2)	.24
Repetition	93,902,535	93,902,535	8.355 (1)	.005
Personality type×message type	13,568,399	6,784,200	0.604 (2)	.55
Personality type×repetition	38,011,469	38,011,469	3.382 (1)	.07
Message type×repetition	3,427,468	1,713,734	0.152 (2)	.86
Personality type×message type×repetition	12,183,242	6,091,621	0.542 (2)	.85
Residuals	865,416,174	11,239,171	N/A^b^	N/A

^a^R^2^=0.28; adjusted R^2^=0.20.

^b^N/A: not applicable.

#### Personality Main Effect

From Figure 2, we can see that independent of other variables, people with a feeling personality type were significantly more likely to adhere to exercise plans. This is an interesting finding that suggests that the effectiveness of mobile apps on influencing behavior might be different for people with different personality types. Two-sample *t* test of mean difference of the dependent variable by personality types shows that the mean of the group with thinking type personality (mean 71.65%, SD 34.24%) is significantly lower (95% CI −31.28 to −5.03; *P*=.007) than that of the feeling type personality group (mean 89.79%, SD 16.39%).

#### Repetition Main Effect

Figure 3 shows that participants who received repeated messages had significantly increased adherence to exercise plans. Two-Sample *t* test of mean difference of the dependent variable by repetition shows that the mean of the group with one more notification (mean 86.12%, SD 20.92%) is significantly higher (95% CI 7.78-31.56; *P*=.001) than that of the group without repetition (mean 63.38%, SD 38.33%).

#### Interaction Between Personality Type and Repetition

We then investigated the significant interaction between personality type and repetition, as reported in Table 3 and Figure 4. The results suggested that (1) when there is no repetition, people with a feeling personality type have a significantly higher completion rate than people with a thinking personality type and, (2) when there is repetition, the completion rate of people with a thinking personality type significantly improves, there is no significant change in the completion rate of people with a feeling personality type, and the 2 groups have no significant difference in terms of completion rate. This is an interesting finding; combined with a previous finding, it suggests that although people with a thinking personality type might have less commitment to the plans they set for themselves, with repeated reminders and messages, these people can achieve a similar completion rate as those with a feeling personality type. This might be because the *mere exposure* effect impacts a person with a thinking personality type more than a person with a feeling personality type.

**Table 3:** Comparison of personality type and repetition groups based on estimated marginal means.

Group compared	Mean difference (%)	*P* value	95% CI
Thinking, repetition-thinking, no repetition	27.34	.001	8.95 to 45.73
Feeling, repetition-feeling, no repetition	−0.83	.99	−31.41 to 29.75
Feeling, no repetition-thinking, no repetition	33.21	.01	5.31 to 61.1
Feeling, repetition-thinking, repetition	5.03	.93	−17.21 to 27.28
Feeling, repetition-thinking, no repetition	32.38	<.001	9.65 to 55.10
Thinking, repetition-feeling, no repetition	−5.86	.94	−33.38 to 21.64

### Summary of Results

Our results suggest that mHealth apps can be effective in promoting adherence. People with a feeling personality type were more likely to adhere to exercise plans than those with a thinking personality type. When receiving repeated reminders and messages, the adherence rates of people with a thinking personality type significantly improved and showed no significant difference from those of people with a feeling personality type. These results are consistent with prior studies that suggest that different personality traits are linked to certain behavioral tendencies, such as proneness to addiction [73] and excessive use of the internet [74]. Our study suggests that personality can contribute to the persistence of adherence to exercise plans.

### Additional Findings

Although the purpose of this research was to examine the effects of tailored messages based on personality and repetition, other tailoring criteria might be selected to motivate exercise behaviors. For example, demographic tailoring has been found to have effects that are independent of theory-based tailoring [75,76]. To further enhance our understanding of the effects of message tailoring and justify the applicability of our model, we conducted an additional analysis to investigate the interactions between sex and message type. We found that emotional messages to women and logical messages to men had a 35.56% higher adherence level (*t*_46_=3.278; *P*=.002) than the other way around (emotional messages to men and logical messages to women). These findings warrant further analysis.

### Robustness Check

In the behavior research field, the issue of violation of parametric tests’ statistical assumptions is rather common, such as skewed distribution, heteroskedasticity, and violation of the independence of errors. Consequently, nonparametric tests have been proposed and widely used to address these issues because of their advantage of not being limited to the assumptions of distribution or homogeneity and because they can be applied to a small sample size [77]. Resampling methods, permutation (randomization test), and bootstrapping are common nonparametric methods.

Permutation tests use all possible permutations of a treatment variable or dependent variable, whereas all other independent variables are fixed to construct the exact null distribution using the available data to determine how extreme the observed test statistic of research interest is against the null distribution. The randomization test [78] relies on the same idea as permutation tests; however, these tests compare the observed statistic against the approximate null distribution generated by repeating a large number of permutations (eg, 10,000-time default in the R package) rather than all possible permutations. As the null distribution is generated empirically from the observed samples and makes no assumptions regarding the population, permutation (randomization test) is beneficial for evaluating the statistical significance or treatment effect of any variable of interest. Another resampling method, which is bootstrap, repeats the available sample and draws from the same sample with replacement to calculate the test statistics and construct an empirical distribution. In experiments, the bootstrap method is a useful way to examine the treatment effects of a designed experiment [79,80].

We used a randomization test and bootstrap resampling method to assess the main effects of the 3 factors and their interactions. By permuting 10,000 times in the randomization test and resampling with replacement 10,000 times for the bootstrap test, our robustness check of the treatment effects shows that the 2 main effects of personality and repetition and the interaction effect between personality and repetition were significant. The results are summarized in Table 4.

**Table 4:** Comparison of resampling methods.

Type of treatment	Base model^a^, *P* value	Randomization test, *P* value	95% bootstrap CI
Emotional message	.24	.14	−17.63 to 55.49
No message	.24	.16	−56.63 to 30.22
Personality	.06	.03	0.07 to 77.89
Repetition	.02	.01	0.85 to 74.48
Emotional message×personality	.65	.30	−83.03 to 17.73
No message×personality	.88	.45	−61.64 to 46.59
Emotional message×repetition	.23	.13	−65.14 to 13.05
No message×repetition	.99	.51	−49.51 to 45.07
Personality×repetition	.06	.06	−91.90 to −6.79
Emotional message×personality×repetition	.42	N/A^b^	−9.81 to 81.93
No message×personality×repetition	.57	N/A	−36.88 to 85.84

^a^Base message type in the regression model is a logical message.

^b^N/A: not applicable.

We also used a randomization test and bootstrap resampling method to examine the effect of *sex-message tailoring*. The effect was significant, with a 95% CI between 17.07% and 55.74% and *P*=.004 in the randomization test. Both resampling methods show consistent results in terms of the treatment effect.

## Discussion

### Learning With ADR

**Figure 5:**
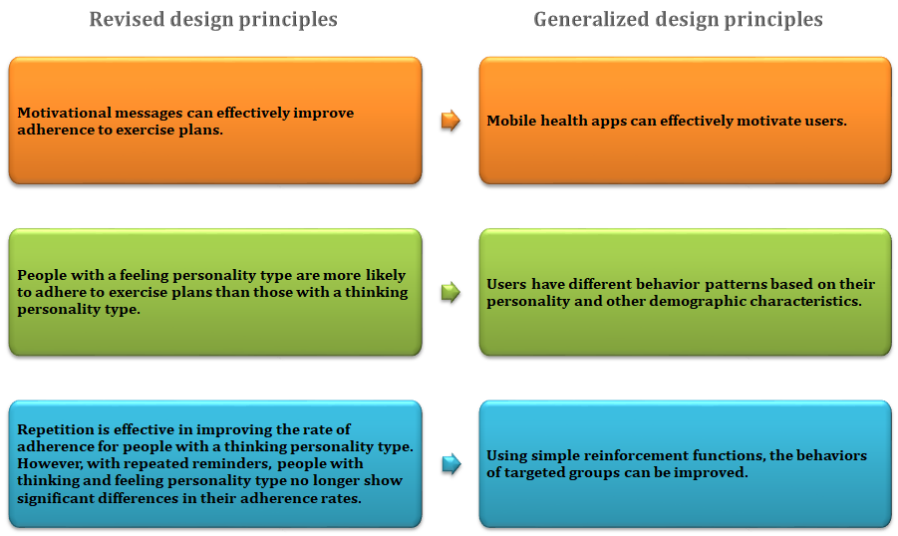
Revised design principles and generalizations.

According to our research process indicated in Figure 1, the evidence collected from the field experiment allowed us to summarize the insights presented in Figure 5. The design principles were revised based on the knowledge from the design and experimental effort and are described in Figure 5. These design principles were used to generate a series of app development problems, such as motivation for other behavioral changes and apps adapted to individual differences. These generalized principles are also presented in Figure 5.

### Research Implications

In this research, we enhanced the ADR framework by developing the ActiveTrack iOS app and incorporating a behavior experiment into the process. When participants downloaded the app, they were randomly assigned to one of the groups for the behavioral experiment. On the basis of the users’ real-time responses, we revised the functions and designs of the app. As a result of the field experiment, we updated the design principles through the ADR process. Our field experiment results suggested that (1) the mobile app was effective in motivating adherence to exercise plans among users with both thinking and feeling personality types, (2) personality type was associated with the likelihood of adherence to exercise plans, and (3) repetition improved the exercise plan adherence rate of people with a thinking-type personality, and with repeated reminders, the adherence rate of people with a thinking personality type becomes similar to that of people with a feeling personality type. Our additional findings further imply that users’ behavior may depend on their (1) job types, (2) exercise habits, and (3) gender. By adding the behavior experiment as a component of ADR, we were able to learn design principles from the behavior experiment results and generalize these principles to a class of design problems: how to effectively motivate users through the personalized design of apps.

This study makes several contributions to the field. First, this research contributes to the literature on mHealth by investigating the effectiveness of mobile apps in motivating health behaviors. Second, this research improves our understanding of the impact of individual personality, message type, and repetition on the effectiveness of motivation. Specifically, through the field experiment, we were able to follow the participants and capture actual behavior change over time, which has been a challenge in behavior-related studies [81]. Third, field surveys, behavior intervention experiments, and the design science approach were normally used separately in the research. In this study, we used all of the above approaches to address the research questions. Combining these methods enabled us to actively design the app by observing usage data and using the design artifact to carry out the research. Thus, by incorporating a field experiment into the research process, we propose an enhanced ADR process. This research process enables us to better connect research with practical and relevant problems. Finally, the study provides several design principles that can be applied to the design of other mHealth and motivational apps, such as apps for lifestyle management, energy-saving behaviors, and adherence-to-treatment plans.

### Lessons Learned From the Apple ResearchKit

In this research, we used the Apple ResearchKit to build artifacts. Apple released ResearchKit, an open-source software framework for medical research, in March 2015. The ResearchKit framework comes with some predefined modules commonly used in research procedures, such as informed consent, surveys, and active tasks, to make the development of mobile apps feasible and more convenient for research. This framework has gained much attention from research institutions and companies owing to its global release [82-84]. However, using ResearchKit and similar development frameworks is still a novel method for scientific research projects, and there are many challenges in conducting research using this method. Through this project, we learned that some of those challenges include difficulty promoting the app because of the number of apps available in the same category in the Apple store, verifying the validity of inputs, and ensuring data security and privacy. In future research, some of these challenges can be overcome during the study design phase. For example, using the heart rate module, the app could more accurately estimate exercise completion. If a study targets a specific group of users, such as people with certain types of disease, it would be a good practice to promote the use of the app among targeted audiences, such as online patient support groups. It is convenient to download the app from the app store, so the barrier for a targeted population to use the app is low regardless of their physical locations.

### Limitations and Future Research

This study has several limitations that can be addressed and improved in future studies. First, the sample size of this research was relatively small because of the difficulty in recruiting active users over the 2 months. However, the sample size was in line with that of a similar study. To address this issue, we used both a randomization test and bootstrap resampling methods to test our treatment effects. The consistent results showed that the main effects of personality and repetition and the two-way interaction between personality and repetition were statistically significant. Future researchers can collaborate with developers, health care providers, and health social networks to increase the potential of participant recruitment. Second, self-reported adherence might be biased. Although we implemented strategies such as reminding participants that the data would not be shared with anyone and honest responses would improve the design of the messages, the validity of self-reported exercise levels could not be verified. Some smartphone features, such as the tracking of heartbeats, can be used for more accurate estimates of exercise in future studies. Third, we attempted to limit the impacts of other confounding factors that may influence the behavior of the users through random assignment to groups; however, there might still have been influences from confounding factors. This is a difficulty that similar research has always faced, as it is difficult to make behavior change in such experiments totally independent of external factors [81].

Potential topics for future research include other strategies for personalized app design and motivation, such as using different theory-based tailoring methods, testing strategies to retain users by analyzing their churn behavior, and identifying ways to use the data collected from these apps to promote health and well-being (ie, different metrics to measure users’ lifestyle change). For example, we found that 14% (12/84) of the users achieved higher average exercise frequencies than their original exercise habits after 2 months. This finding implies that users may change their lifestyles using our mHealth app. Future research can also focus on the clinical validation of the experimental results, ways of using economic incentives to promote healthy lifestyles, new business models for health care providers and insurance companies to motivate adherence, and the use of other wearable devices, such as smartwatches, to motivate health behaviors.

## Appendix

Multimedia Appendix 1 Emotional and logical messages.

Multimedia Appendix 2 Screenshots of the design artifact—ActiveTrack.

Multimedia Appendix 3 Consent form declaration.

Multimedia Appendix 4 Questionnaire for demographics information.

Multimedia Appendix 5 Myers-Briggs Type Indicator assessment.

## Abbreviations

ADR: action design research
MBTI: Myers-Briggs Type Indicator
mHealth: mobile health

## References

[1] Physical activity. U.S. Department of Health and Human Services.

[2] Booth F, Roberts C, Laye M (2012). Lack of exercise is a major cause of chronic diseases. Compr Physiol.

[3] Mokdad AH, Marks JS, Stroup DF, Gerberding JL (2004). Actual causes of death in the United States, 2000. J Am Med Assoc.

[4] Park A (2012). Lack of exercise as deadly as smoking, study finds. Time.

[5] Coulson J, McKenna J, Field M (2008). Exercising at work and self‐reported work performance. Intl J of Workplace Health Mgt.

[6] (2015). Patient adoption of mHealth. IMS Institute for Healthcare Informatics.

[7] (2017). The growing value of digital health. The IQVIA Institute Reports.

[8] (2021). Healthcare consumers in a digital transition. Rock Health.

[9] Maddison R, Rawstorn JC, Islam SM, Ball K, Tighe S, Gant N, Whittaker RM, Chow CK (2019). mHealth interventions for exercise and risk factor modification in cardiovascular disease. Exerc Sport Sci Rev.

[10] Oinas-Kukkonen H, Harjumaa M (2008). Towards deeper understanding of persuasion in software and information systems. Proceedings of the First International Conference on Advanced in Computer-Human Interaction.

[11] Tomlinson M, Rotheram-Borus MJ, Swartz L, Tsai AC (2013). Scaling up mHealth: where is the evidence?. PLoS Med.

[12] Marcano BJ, Huckvale K, Greenfield G, Car J, Gunn LH (2013). Smartphone and tablet self management apps for asthma. Cochrane Database Syst Rev.

[13] Payne HE, Lister C, West JH, Bernhardt JM (2015). Behavioral functionality of mobile apps in health interventions: a systematic review of the literature. JMIR Mhealth Uhealth.

[14] Cui M, Wu X, Mao J, Wang X, Nie M (2016). T2DM self-management via smartphone applications: a systematic review and meta-analysis. PLoS One.

[15] Oinas-Kukkonen H, Harjumaa M (2009). Persuasive systems design: key issues, process model, and system features. Commun Assoc Inf Syst.

[16] Geuens J, Swinnen TW, Westhovens R, de VK, Geurts L, Vanden AV (2016). A review of persuasive principles in mobile apps for chronic arthritis patients: opportunities for improvement. JMIR Mhealth Uhealth.

[17] Middelweerd A, Mollee JS, van der Wal CN, Brug J, Velde SJ (2014). Apps to promote physical activity among adults: a review and content analysis. Int J Behav Nutr Phys Act.

[18] Matthews J, Win KT, Oinas-Kukkonen H, Freeman M (2016). Persuasive technology in mobile applications promoting physical activity: a systematic review. J Med Syst.

[19] Maibach EW, Weber D, Massett H, Hancock GR, Price S (2006). Understanding consumers' health information preferences: development and validation of a brief screening instrument. J Health Commun.

[20] Peppers D, Rogers M, Dorf B (1999). Is your company ready for one-to-one marketing?. Harv Bus Rev.

[21] Storey J, Saffitz G, Rimon J, Glanz K, Rimer BK, Viswanath K (2008). Social marketing. Health Behavior and Health Education: Theory, Research and Practice. 4th Ed.

[22] Strecher V, Rimer B, Monaco K (1989). Development of a new self-help guide--Freedom From Smoking for you and your family. Health Educ Q.

[23] Robinson RG, Sutton CD, James DA, Orleans CT Pathways to freedom: Winning the fight against tobacco. US Department of Health and Human Services, Centers for Disease Control and Prevention.

[24] Rimer B, Orleans C, Fleisher L, Cristinzio S, Resch N, Telepchak J, Keintz M (1994). Does tailoring matter? The impact of a tailored guide on ratings and short-term smoking-related outcomes for older smokers. Health Educ Res.

[25] Davis SW, Cummings KM, Rimer BK, Sciandra R, Stone JC (1992). The impact of tailored self-help smoking cessation guides on young mothers. Health Educ Q.

[26] Keintz MK, Fleisher L, Rimer BK (1994). Reaching mothers of preschool-aged children with a targeted quit smoking intervention. J Community Health.

[27] Jung CG, Read H, Fordham M, Adler G, Hull RF (1992). Psychiatric studies. The Collected Works of CG Jung, Vol. 1. 2nd Edition.

[28] Myers IB, McCaulley MH, Quenk NL, Hammer AL (1998). MBTI Manual: A Guide to the Development and Use of the Myers-Briggs Type Indicator, 3rd Edition.

[29] Graff WS (1976). The effectiveness of systematic desensitization in the reduction of test anxiety in Jungian thinking versus feeling personality types. ProQuest Information & Learning.

[30] Carskadon TG (1979). Clinical and counseling aspects of the Myers-Briggs Type Indicator: a research review. Research in Psychological Type.

[31] Jinkerson J, Masilla A, Hawkins RC (2015). Can MBTI dimensions predict therapy outcome: differences in the thinking-feeling function pair in CBT. Res Psychothe Psychopathol Proc Outcome.

[32] Arain AA (1967). Relationships among counseling clients' personalities, expectations,problems (Doctoral dissertation). Rutgers University, New Brunswick, NJ.

[33] Garden A (1997). Relationships between MBTI profiles, motivation profiles, and career paths. J Psychol Type.

[34] DeBono KG, Harnish RJ (1988). Source expertise, source attractiveness, and the processing of persuasive information: a functional approach. J Pers Soc Psychol.

[35] Klein WM, Harris PR (2009). Self-affirmation enhances attentional bias toward threatening components of a persuasive message. Psychol Sci.

[36] Singh SN, Cole CA (2018). The effects of length, content, and repetition on television commercial effectiveness. J Mark Res.

[37] Malaviya P (2007). The moderating influence of advertising context on ad repetition effects: the role of amount and type of elaboration. J Consum Res.

[38] Lewis IM, Watson B, White KM, Tay R (2007). Promoting public health messages: should we move beyond fear-evoking appeals in road safety?. Qual Health Res.

[39] Gifford R, Comeau LA (2011). Message framing influences perceived climate change competence, engagement, and behavioral intentions. Glob Environ Change.

[40] Prentice-Dunn S, Floyd D, Flournoy JM (2001). Effects of persuasive message order on coping with breast cancer information. Health Educ Res.

[41] Sherman DK, Mann T, Updegraff JA (2006). Approach/avoidance motivation, message framing, and health behavior: understanding the congruency effect. Motiv Emot.

[42] Prestwich A, Perugini M, Hurling R (2009). Can the effects of implementation intentions on exercise be enhanced using text messages?. Psychol Health.

[43] Quilici J, Fugon L, Beguin S, Morange PE, Bonnet J, Alessi M, Carrieri P, Cuisset T (2013). Effect of motivational mobile phone short message service on aspirin adherence after coronary stenting for acute coronary syndrome. Int J Cardiol.

[44] Pallak SR, Murroni E, Koch J (1983). Communicator attractiveness and expertise, emotional versus rational appeals, and persuasion: a heuristic versus systematic processing interpretation. Soc Cogn.

[45] Miceli M, Rosis FD, Poggi I (2006). Emotional and non-emotional persuasion. Appl Artif Intell.

[46] Tykocinskl O, Higgins ET, Chaiken S (2016). Message framing, self-discrepancies, and yielding to persuasive messages: the motivational significance of psychological situations. Pers Soc Psychol Bull.

[47] Shen L, Dillard JP (2009). Message frames interact with motivational systems to determine depth of message processing. Health Commun.

[48] Toulmin SE (2003). The Uses of Argument.

[49] Kim D, Benbasat I (2006). The effects of trust-assuring arguments on consumer trust in internet stores: application of toulmin's model of argumentation. Inf Syst Res.

[50] Clore GL, Gasper K (2000). Feeling is believing: Some affective influences on belief. Emotions and Beliefs.

[51] Murphy ST, Zajonc RB (1993). Affect, cognition, and awareness: affective priming with optimal and suboptimal stimulus exposures. J Pers Soc Psychol.

[52] Cacioppo JT, Petty RE (1979). Effects of message repetition and position on cognitive response, recall, and persuasion. J Pers Soc Psychol.

[53] Burgoon M, Miller MD (2016). Overcoming resistance to persuasion VIA contiguous reinforcement and repetition of message. Psychol Rep.

[54] Zajonc R (2016). Mere exposure: a gateway to the subliminal. Curr Dir Psychol Sci.

[55] Saegert SC, Jellison JM (1970). Effects of initial level of response competition and frequency of exposure on liking and exploratory behavior. J Pers Soc Psychol.

[56] Johnson HH, Watkins TA (2013). The effects of message repetitions on immediate and delayed attitude change. Psychon Sci.

[57] Weiss RF (1971). Role playing and repetition effects on opinion strength. J Soc Psychol.

[58] McCullough JL, Ostrom TM (1974). Repetition of highly similar messages and attitude change. J Appl Psychol.

[59] Smith GF, Dorfman DD (1975). The effect of stimulus uncertainty on the relationship between frequency of exposure and liking. J Pers Soc Psychol.

[60] Harrison A (1977). Mere exposure. Adv Exp Soc Psychol.

[61] Bettinghaus EP (1986). Health promotion and the knowledge-attitude-behavior continuum. Prev Med.

[62] Brooks ME, Highhouse S (2006). Familiarity breeds ambivalence. Corp Reputation Rev.

[63] Fang X, Singh S, Ahluwalia R (2007). An examination of different explanations for the mere exposure effect. J Consum Res.

[64] Cook C, Heath F, Thompson RL (2016). A meta-analysis of response rates in web- or internet-based surveys. Educ Psychol Meas.

[65] Pohl RF (2005). Cognitive Illusions: A Handbook on Fallacies and Biases in Thinking, Judgement and Memory, First Edition.

[66] Serenko A, Bontis N (2011). What's familiar is excellent: The impact of exposure effect on perceived journal quality. J Informetr.

[67] Malone TW (1983). How do people organize their desks?. ACM Trans Inf Syst.

[68] Lord S, Ward J, Williams P, Strudwick M (1995). The effect of a 12-month exercise trial on balance, strength, and falls in older women: a randomized controlled trial. J Am Geriatr Soc.

[69] Estabrooks P, Carron A (1999). Group cohesion in older adult exercisers: prediction and intervention effects. J Behav Med.

[70] Sein MK, Henfridsson O, Purao S, Rossi M, Lindgren R (2011). Action design research. MIS Q.

[71] Cohen J (2016). A coefficient of agreement for nominal scales. Educ Psychol Meas.

[72] Free C, Phillips G, Galli L, Watson L, Felix L, Edwards P, Patel V, Haines A (2013). The effectiveness of mobile-health technology-based health behaviour change or disease management interventions for health care consumers: a systematic review. PLoS Med.

[73] Müller SE, Weijers H, Böning J, Wiesbeck GA (2008). Personality traits predict treatment outcome in alcohol-dependent patients. Neuropsychobiology.

[74] Munno D, Cappellin F, Saroldi M, Bechon E, Guglielmucci F, Passera R, Zullo G (2017). Internet addiction disorder: personality characteristics and risk of pathological overuse in adolescents. Psychiatry Res.

[75] Noar SM, Harrington NG, Aldrich RS (2016). The role of message tailoring in the development of persuasive health communication messages. Annal Int Commun Asso.

[76] Harrington NG, Noar SM (2012). Reporting standards for studies of tailored interventions. Health Educ Res.

[77] Peres-Neto PR, Olden JD (2001). Assessing the robustness of randomization tests: examples from behavioural studies. Anim Behav.

[78] Edgington ES, Lovric M (2014). Randomization tests. International Encyclopedia of Statistical Science.

[79] Davison AC, Hinkley DV (1997). Bootstrap Methods and Their Application (Cambridge Series in Statistical and Probabilistic Mathematics).

[80] Kenett RS, Rahav E, Steinberg DM (2006). Bootstrap analysis of designed experiments. Qual Reliab Engg Int.

[81] Andrews PY (2012). System personality and persuasion in human-computer dialogue. ACM Trans Interact Intel Syst.

[82] Eisenstein M (2015). GSK collaborates with Apple on ResearchKit. Nat Biotechnol.

[83] Webster DE, Suver C, Doerr M, Mounts E, Domenico L, Petrie T, Leachman SA, Trister AD, Bot BM (2017). The Mole Mapper Study, mobile phone skin imaging and melanoma risk data collected using ResearchKit. Sci Data.

[84] Zens M, Woias P, Suedkamp NP, Niemeyer P (2017). "Back on Track": a mobile App observational study using Apple's ResearchKit framework. JMIR Mhealth Uhealth.

