# Supplementary material for: Motivating Adherence to Exercise Plans Through a Personalized Mobile Health App: Enhanced Action Design Research Approach
Source: JMIR Mhealth Uhealth. 2021 Jun 2;9(6):e19941. doi: 10.2196/19941 (PMC8209532; doi:10.2196/19941)
Supplement: Multimedia Appendix 1 [file mhealth_v9i6e19941_app1.docx]

| No. | Emotional Messages | No. | Logical Messages |
| --- | --- | --- | --- |
| E1 | Nothing feels better than a finished workout. | L1 | Research shows that even one session of exercise will enhance positive mood. |
| E2 | A bad day can be made better by going to the gym. | L2 | Research show that even one session of exercise enhances creative thinking. |
| E3 | Good things come to those who sweat. | L3 | Literature suggests that creative people use bodily movement to help overcome thinking blocks. |
| E4 | We don’t know how strong we are until being strong is our only choice. | L4 | "Exercise is the single best thing you can do for your brain in terms of mood, memory, and learning." – US News and World Report. |
| E5 | Today, I will love myself enough to exercise. | L5 | The one habit nearly all highly successful people seem to have in common is exercise. |
| E6 | Think about how good you will feel when you are done with the workout. | L6 | Barack Obama, president of the United States, exercises for 45 minutes a day, six days per week. |
| E7 | The only bad workout is the one that didn’t happen. | L7 | Mark Zuckerberg, co-founder and CEO of Facebook, works out at least three days per week. |
| E8 | Sweat today, and smile tomorrow. | L8 | The American Heart Association recommends at least 150 minutes per week of moderate exercise or 75 minutes per week of vigorous exercise. |
| E9 | No goal was ever met without a little sweat. | L9 | The American Heart Association recommends exercising thirty minutes a day, five times a week to improve overall cardiovascular health. |
| E10 | You are strong, fit and fabulous! | L10 | Research suggests that exercise improves your learning ability and grow brain cells. |
| E11 | You are only one workout away from a good mood. | L11 | Doctors recommend using exercise to alleviate stress, anger, anxiety, and depression. |
| E12 | Wake up with determination, go to bed with satisfaction! | L12 | Studies show that exercise boosts self-control and social activity. |
| E13 | You are doing a fabulous job! | L13 | It is well established by research that doing regular physical activity can make you feel good about yourself. |
| E14 | You are amazing and beautiful every day! | L14 | Strong scientific evidence shows that physical activity can help you maintain your weight over time. |
| E15 | The best project you will ever work on is you! | L15 | Science shows that physical activity can reduce your risk of dying early from the leading causes of death, like heart disease and some cancers. |
| E16 | Don’t stop until you are proud! | L16 | Research suggests that regular physical activity is one of the most important things you can do for your health. |
| E17 | Build a better, stronger version of yourself! | L17 | Research show that being physically active lowers your risk for two types of cancer: colon and breast cancer. |
| E18 | Make yourself proud! | L18 | Research shows that doing regular physical activity of at least a moderately intense level can slow the loss of bone density that comes with age. |
| E19 | If you are looking for a sign to go workout, this is it. Go now! | L19 | Regular physical activity can help keep your thinking, learning, and judgment skills sharp as you age. |
| E20 | Keeping fit is form of self-respect. | L20 | Research shows that regular exercise can reduce your risk of depression and may help you sleep better. |
| E21 | The body achieves what the mind believes. | L21 | Research shows that you can put yourself at lower risk of dying early by doing at least 150 minutes a week of moderate-intensity aerobic activity. |
| E22 | Take care of your body. It’s the only place you have to live. | L22 | Studies show that 30 minutes of exercise a day in conjunction with a healthy diet can be effective in losing weight. |
| E23 | Get better one workout at a time, one day at a time, one meal at a time! | L23 | For health, doctors "prescribe" at least 30 minutes of moderate exercise or 15 minutes of intense exercise a day. |
| E24 | Be sore today, and become strong tomorrow. | L24 | Studies clearly show that regular exercise helps the heart. |
| E25 | It’s never too late to become a fitter, healthier person. | L25 | Studies show that regular exercise decreases the risk of heart disease, high blood pressure, and stroke. |
| E26 | Exercise for the pure, absolute joy! | L26 | Studies show that exercise can improve sleep quality and help you get a better night’s rest. |
| E27 | Every day is another chance to get stronger! | L27 | Studies show that in general, people who exercise regularly are healthier and get sick less often. |
| E28 | Three months from now you will thank yourself. | L28 | Physical activity stimulates the release of feel-good brain chemicals, including endorphins to help you relieve stress. |
| E29 | Fit is not a destination, it is a way of life. | L29 | Research has found that regular physical activity can increase your confidence. |
| E30 | A one-hour workout is just 4 percent of your day! | L30 | There is scientific evidence that regular physical activity helps you increase energy levels. |
