# Supplementary figures and images for: Motivating Adherence to Exercise Plans Through a Personalized Mobile Health App: Enhanced Action Design Research Approach
Source: JMIR Mhealth Uhealth. 2021 Jun 2;9(6):e19941. doi: 10.2196/19941 (PMC8209532; doi:10.2196/19941)

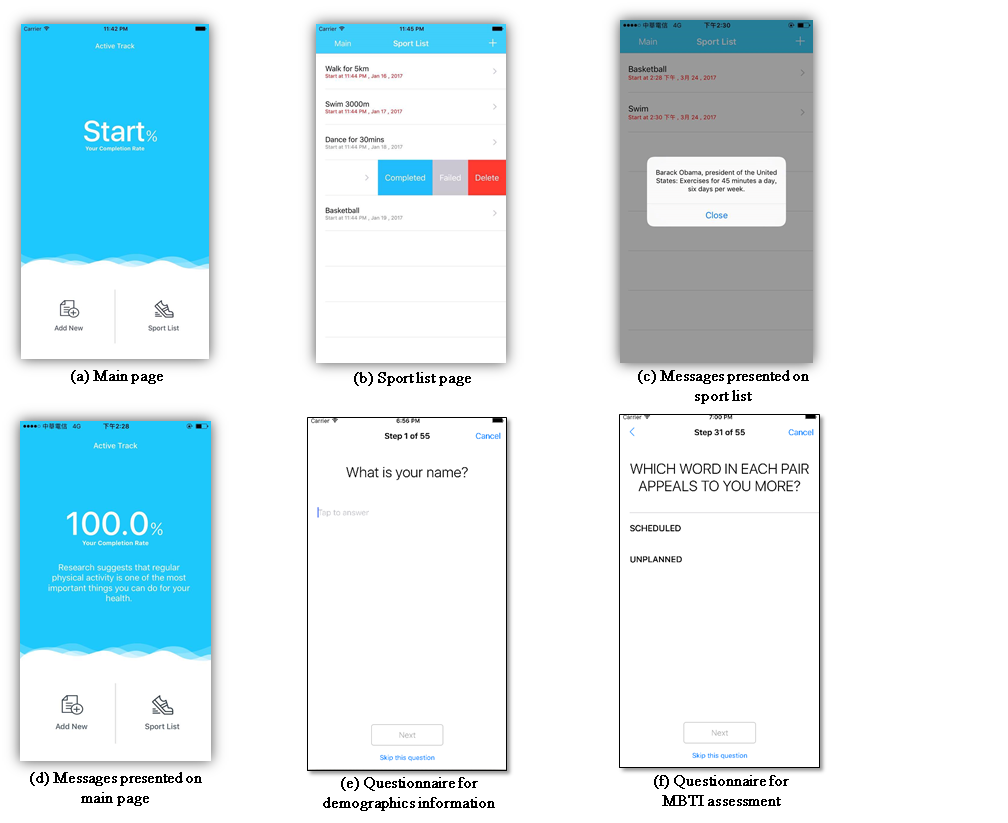

Supplement: Multimedia Appendix 2 [file mhealth_v9i6e19941_app2.png]
