# Supplementary material for: Motivating Adherence to Exercise Plans Through a Personalized Mobile Health App: Enhanced Action Design Research Approach
Source: JMIR Mhealth Uhealth. 2021 Jun 2;9(6):e19941. doi: 10.2196/19941 (PMC8209532; doi:10.2196/19941)
Supplement: Multimedia Appendix 3 [file mhealth_v9i6e19941_app3.pdf]

Use Mobile Application

Purpose: The Department of Business Administration in the University of Illinois at Urbana-Champaign is conducting an experiment investigating motivation based on personality types. The research findings based on this study are expected to yield valuable knowledge relevant to understanding how to motivate people based on their personal differences.

Procedures: You need to be 18 years old or older to participate in the study. Participation in the study is designed to be one month. Your identification information will not be collected.

Voluntary Nature: Your participation is voluntary. You may withdraw from participation at any time without adverse consequences. Your participation ends with the completion of using the application for one month, and no further activity is required. You may continue to use the application if you wish, and no information will be collected after one month.

Confidentiality: None of the data collected will be used to identify particular individuals. There are no questions that require you to provide identifying information, such as your name, address, social security number, etc. All data will be presented in aggregated form only. Your use of this application indicates your consent to participation. However, laws and university rules may require us to disclose individual answers. For example, if required by law or university policy, the study information may be seen or copied by the following people or groups: a) the university committee and office that reviews and approves research studies, the Institutional Review Board (IRB) and Office for Protection of Research Subjects or b) university and state auditors and departments of the university responsible for the oversight of research.

Risks and Benefits: There is no known or expected risk to participants of this study, because we are not collecting your name. This is purely academic research, and the findings of this research might benefit society as this study attempts to shed light on effective ways to motivate people.

Contact: The research team sincerely appreciates your participation in this study. Any questions, concerns or complaints that you may have about this study can be answered by Dr. Wencui Han. She can be reached through the Department of Business Administration, University of Illinois at Urbana Champaign, or by phone at 217-765-4966.

Thank you very much for your participation.
