# Supplementary material for: Motivating Adherence to Exercise Plans Through a Personalized Mobile Health App: Enhanced Action Design Research Approach
Source: JMIR Mhealth Uhealth. 2021 Jun 2;9(6):e19941. doi: 10.2196/19941 (PMC8209532; doi:10.2196/19941)
Supplement: Multimedia Appendix 4 [file mhealth_v9i6e19941_app4.pdf]

Demographic survey questions

1. How old are you?

2. What is your sex?

- Female
- Male

3. What is your race?

- White
- Black or African American
- American Indian
- Alaska Native
- Asian
- Hawaiian Native and Pacific Islander
- Other

4. How many hours do you sit for work per day?

- <4
- 4-8
- 8-12
- >12

5. Which description best describe your job type?

- Professional, manager or administrative work
- Manual work
- Customer interaction, entertainment, sales or other service-oriented work

6. How much do you weight?

7. How many times per week do you exercise?
