# Supplementary material for: Motivating Adherence to Exercise Plans Through a Personalized Mobile Health App: Enhanced Action Design Research Approach
Source: JMIR Mhealth Uhealth. 2021 Jun 2;9(6):e19941. doi: 10.2196/19941 (PMC8209532; doi:10.2196/19941)
Supplement: Multimedia Appendix 5 [file mhealth_v9i6e19941_app5.pdf]

Myer-Briggs Type Indicator questionnaire for thinking and feeling preference by Katharine C. Briggs & Isabel Briggs Myers

Directions:

There are no "right" or "wrong" answers to the questions on this inventory. Your answers will help show how you like to look at things and how you like to go about deciding things. Knowing your own preferences and learning about other peoples' preferences can help you understand what your special strengths are, what type of work you might enjoy and be successful doing, and how people with different preferences can relate to each other and be valuable to society.

Read each question carefully and select one of the two choices given that applies to you by circling either "a" or "b".

<u>Part1: Which answer is closer to telling how you usually feel or act?</u>

1. Do you more often let

A. Your heart rule your head. or

B. Your head rule your heart?

2. Is it a higher compliment to be called

A. A person of real feeling or

B. A consistently reasonable person?

3. Do you usually

A. Value sentiment more than logic, or

B. Value logic more than sentiments?

<u>Part 2:Which word in each pair appeals to you more?</u>

(Think what the word means, not how it looks or sounds)

4.	A. Convincing	B. Touching
5.	A. Benefits	B. Blessings
6.	A. Peacemaker	B. Judge

7. A. Analyze B. Sympathize

8. A. Determined B. Devoted

9. A. Gentle B. Firm

10. A. Justice B. Mercy

11. A. Firm-minded B. Warm hearted

12. A. Feeling B. Thinking

13. A. Foresight B. Compassion

14. A. Hard B. Soft

MBTI Scoring Sheet

Directions:

Corresponding to your choice for each question. Some points are given for a combination of questions and choices. Circle the points in front of your choices. Finally add the circled number points in each category.

Question	Choice	Point	Choice	Point
1	B	2	A	1
2	B	2	A	1
3	B	2	A	2
4	A	2	B	1
5	A	1	B	1
6	B	2	A	0
7	A	1	B	2
8	A	1	B	1
9	B	2	A	1
10	A	1	B	2
11	A	2	B	0
12	B	2	A	1
13	A	2	B	1
14	A	2	B	0
